# Draft Genome Sequence of a Lactobacillus gasseri Strain Isolated from the Catheterized Urine of a Healthy Postmenopausal Woman

**DOI:** 10.1128/mra.00021-22

**Published:** 2022-05-09

**Authors:** James A. Johnson, Jennifer L. Modliszewski, Nazema Y. Siddiqui, Tatyana A. Sysoeva

**Affiliations:** a Department of Biological Sciences, University of Alabama in Huntsville, Huntsville, Alabama, USA; b Duke University Center for Genomic and Computational Biology, Department of Bioinformatics and Biostatistics, Duke University, Durham, North Carolina, USA; c Division of Urogynecology and Reconstructive Pelvic Surgery, Department of Obstetrics and Gynecology, Duke University, Durham, North Carolina, USA; Loyola University Chicago

## Abstract

Urinary microbiome composition has been found to associate with health status and to change with age. Lactobacillus gasseri is one of the most frequently found lactic acid bacteria in the vaginal and urinary tracts of women. Here, we report a draft genome sequence of a urinary L. gasseri strain isolated from a healthy postmenopausal woman.

## ANNOUNCEMENT

Many lactobacilli—lactic acid-forming bacteria—have long been considered human commensals; they are found in abundance in the oral, vaginal, and gastrointestinal tracts of healthy individuals (1–3). Recent studies indicate the presence of these bacteria in the lower urinary tract (the urethra and bladder) (4–6) and note a possible connection between urinary health and the presence of lactobacillus species (2, 4, 7, 8). In particular, Lactobacillus gasseri is one of the four most abundant lactobacilli found in the vaginal and urinary tracts of women. It also was found to be associated with urinary incontinence in one study but not in another (9, 10). No genetic determinants of L. gasseri from different tracts have been identified so far.

In a previously published study by Vaughan and colleagues (2), Lactobacillus strain 5006-2 was isolated using Enhanced Quantitative Urine Culture (6) from the catheterized urine of an asymptomatic postmenopausal 66-year-old female patient. Using mass spectrometry (MALDI-TOF), the strain was typed as a group Lactobacillus gasseri/acidophilus. The strain was cultured on blood tryptic soy plates and grew robustly in liquid Man Rogosa Sharpe (MRS) broth at 35°C without agitation. The stock strain was preserved in 14% (wt/vol) glycerol stock at −80°C. To obtain more details, we sequenced the whole genome of the isolate. For that purpose, genomic DNA was isolated using the Qiagen UltraClean kit and sequenced using an Illumina platform (NextSeq; 150-bp paired-end reads) at the Sequencing and Genomic Technologies Core Facility of the Duke University Center for Genomic and Computational Biology. The sequencing produced a total of 15.8 million reads. The raw paired-end read sequences were trimmed of adapter and low-quality sequences using the Trim Galore v0.4.4 toolkit (11) with default settings, which employs Cutadapt v1.16 (12). The quality of the raw reads was assessed using FastQC v0.11.7 (13), and the genomes were assembled using Unicycler v0.4.4 (14). To evaluate the assembly statistics and completeness, QUAST v4.5 (15) and BUSCO v3.0.2 (16) were used to search for conserved single-copy orthologs using the most precise species-appropriate database available—lactobacillales_odb9. The strain was identified as Lactobacillus gasseri. To ensure the absence of contamination in the final assembly, command line blastn (BLAST+ v2.7.1) (17) was run in MegaBLAST mode against the nucleotide (nt) database. The L. gasseri 5006-2 assembly is an approximately 1.79-Mbp genome with a GC content of 34.9%. The assembly consists of 21 contigs with maximum and minimum lengths of 955,358 bp and 124 bp, respectively, and an *N*_50_ value of 955,358 bp. Only the 16 contigs larger than 200 bp were deposited at GenBank.

The draft genome was uploaded and annotated using the automated Prokaryotic Genome Annotation Pipeline (PGAP) v5.1 (18). To determine whether L. gasseri 5006-2 can produce bacteriocins, we used BAGEL4 (19), which identified a putative cluster for helveticin J (20) homolog production. No CRISPR/Cas loci were detected in the assemblies using CRISPRCasFinder (21). BLAST searches for the new lactobacilli genome identified three closely related strains: L. gasseri strains ATCC 33323 (22), DSM 14869 (23), and 4M13 (24). While the source of human ATCC 33323 type isolate is unclear, the DSM 14869 strain was isolated from the vagina, and 4M13 was isolated from infant feces.

No plasmids were identified in L. gasseri 5006-2 using PlasmidFinder 2.1 (25), possibly due to the poor knowledge of lactobacilli plasmids in general and in urinary isolates in particular (26, 27). An examination of assembly graphs using BANDAGE v0.8.1 (28) and the BLAST search results showed the presence of at least one plasmid approximately 40 kB long with a copy number of ~2.2 to 2.4. The BLAST results showed similarity with plasmids pEB01-1 and pEB01-2 (GenBank accession numbers CP008839.1 and CP008840.1) found in the above-mentioned strain DSM 14869, as well as several unpublished plasmids from other L. gasseri isolates (CP044414.1, CP087762.1, and CP072658.1). The plasmid-related contigs encode plasmid replication protein A, toxin/antitoxin pairs, recombination enzymes, and transporters.

### Data availability.

The draft genome assembly for L. gasseri strain 5006-2 has been deposited at GenBank under the accession number JAGEKM000000000.1 and the raw sequencing data at the Sequence Read Archive under the accession number SRR13983850.
